# Influence of Foliar Application of Nanoparticles on Low Temperature Resistance of Rice Seedlings

**DOI:** 10.3390/plants13212949

**Published:** 2024-10-22

**Authors:** Shafi Ullah, Muhammad Ikram, Jian Xiao, Atika Khan, Ismail Din, Jianliang Huang

**Affiliations:** 1 National Key Laboratory of Crop Genetic Improvement, Ministry of Agriculture Key Laboratory of Crop Ecophysiology and Farming System in the Middle Reaches of the Yangtze River, College of Plant Science and Technology, Huazhong Agricultural University, Wuhan 430070, China; shafi@aup.edu.pk (S.U.); xiaoj202410@163.com (J.X.); atkakhan7@gmail.com (A.K.); 2 MOA Key Laboratory of Crop Ecophysiology and Farming System in the Middle Reaches of the Yangtze River, College of Plant Science and Technology, Huazhong Agricultural University, Wuhan 430070, China; r4rana88@gmail.com; 3 National Key Laboratory of Crop Genetic Improvement, Huazhong Agricultural University, Wuhan 430070, China; ismaildin@webmail.hzau.edu.cn

**Keywords:** foliar application, rice, nanoparticles, chlorophyll, antioxidant

## Abstract

Chilling stress, a common abiotic factor, adversely affects the growth and biomass of rice seedlings during the early stages, ultimately reducing the yield. Effective strategies to mitigate these negative impacts are essential for improving rice productivity. The application of nanotechnology in agriculture, particularly nanoparticles (NPs), has shown a promising effect in alleviating chilling stress in plants. This study evaluates the effects of various nanoparticles, ZnO (0, 50, 100, and 200 mg/L), Fe_2_O_3_ (0, 50, 75, and 100 mg/L), TiO_2_ (0, 50, 75, and 100 mg/L), and CeO_2_ (0, 50, 75, and 100 mg/L) on the chilling resistance with one control (a water spray) under a normal temperature. Four rice cultivars: LLY-7108 and XZX-6 (Low-temperature-tolerant), and LLY-32 and ZJZ-17 (Low-temperature-susceptible) were tested in this experiment. Rice seedlings were subjected to low temperature conditions (12 h light 14 °C/12 h dark, at 10 °C) for five days, followed by seven days of recovery. The results of this study demonstrate that NPs significantly enhanced seedling height fresh/dry weight and root length compared to untreated controls under chilling stress. NP treatment also reduced the reactive oxygen species (ROS), malondialdehyde (MDA), and proline content, while enhancing superoxide dismutase (SOD), peroxidase (POD), and catalase (CAT) activities, thereby mitigating oxidative damage. The four rice varieties exhibited clear signs of rapid growth recovery and positive physiological changes due to NPs’ application. Among the tested cultivars, LLY-7108 showed the most substantial recovery and physiological responses, while ZJZ-17 exhibited the least. The findings of this study indicate that the foliar application of ZnO (100 mg/L), Fe_2_O_3_ (50 mg/L), TiO_2_ (50 mg/L), and CeO_2_ (75 mg/L) NPs effectively mitigates chilling stress in rice seedlings, likely by enhancing the antioxidant enzymatic activity while reducing the oxidative damage. This study highlights the potential of NPs as effective agents in reducing the adverse effects of chilling stress on rice.

## 1. Introduction

Rice (*Oryza sativa* L.) is an important cereal crop and serves as the staple food for over half of the world’s population [1]. Low-temperature problems are widespread in the major rice-producing regions worldwide, including the United States, Japan, Korea, and China. In hilly areas of tropical regions and in the temperate zones where rice is cultivated, chilling damage is a primary constraint in increasing rice production b [2,3]. Direct-seeded early indica rice is often more exposed to lower temperatures at seedling stages than transplanted rice because of global warming and frequently occurring extreme weather [4]. The direct seeding of early indica rice followed by heavy rainfall and a “cold spell in late spring” destroyed a significant number of seeds, resulting in uneven seedling emergence and poor population growth, ultimately reducing the yield of direct-seeded rice [5]. In 2010, a “cold spell in later spring” affected the double season early rice in the middle and lower reaches of the Yangtze River, causing extensive damage to direct-seeded rice fields with rotten seeds and shoots. This had a severe impact on the production of direct-seeded rice [6]. Low-temperature stress is a common abiotic environmental condition in crop production that results in oxidative stress through the excessive generation of reactive oxygen species (ROS), ultimately leading to membrane lipid peroxidation, a loss of cell membrane integrity, and the induction of several abnormal physiological processes [7]. APX ascorbate peroxide (APX) and glutathione (GSH) are cold stable antioxidant enzymes that scavenge excessive ROS in cold-protecting processes in plant [8]. Exposure to low temperatures adversely affects the activity of photosynthetic enzymes, the concentration of chlorophyll, and several physiological and biochemical processes in rice [9]. A low temperature decreases both the photosynthetic dark reaction activity and light reaction activity, leading to a decrease in the light energy transfer efficiency and light energy conversion efficiency [10]. The final product of sugar consumption is CO_2_, which plays a crucial role in the regulation of mitochondrial metabolism and growth balance [11] due to its coupling with pyruvate dehydrogenase (PDH), α-keto-glutarate dehygenease (α-KGDH), in a tricarboxylic acid (TCA)-cycle.

The minimization of chilling stress through various ways is vital for the productivity of rice. Nanoparticles (NPs) are an efficient tool for reducing the impact of abiotic stresses [12]. Numerous metal oxide nanoparticles (MONPs), such as titanium dioxide (TiO_2_), iron oxide (Fe_3_O_4_), zinc oxide (ZnO), and CeO_2_, have gained considerable attention in recent years due to their environmentally favorable use in agriculture. Metal oxide nanoparticles gained significant interest in various fields due to their unique physical and chemical properties, which are distinct from their bulk counterparts, and their small size, which provides a large surface-area-to-volume ratio, making them more effective in various field [13]. MONPs have been utilized to protect plants from oxidative stress by increasing the activity of antioxidant enzymes such as superoxide dismutase (SOD), catalase (CAT), and peroxidase (POX) [14]. They have the potential to reduce the detrimental impact of drought on plant physiological functions by lowering the malondialdehyde (MDA) and hydrogen peroxide (H_2_O_2_) contents and maintaining photosynthetic systems [14,15]. Under abiotic stress, they play a role in signaling pathways, defense, metabolism, and regulatory activities. For example, TiO_2_ nanoparticles (TiO_2_NPs) decreased oxidative damage and lipid peroxidation in response to drought stress, as shown by reduced H_2_O_2_ and MDA concentrations [16]. MONPs can penetrate chloroplasts and react with the photosystem II reaction center, hence increasing electron transmission, oxygen evolution, and light absorption in chloroplasts under drought-induced oxidative stress [17]. The utilization of TiO_2_ nanoparticles not only mitigated the harm to the membrane caused by cold stress, but also avoided oxidative stress in chickpeas [18]. In addition, the TiO_2_ treatment increased crop cold tolerance by stabilizing carotenoid and chlorophyll accumulation, increasing superoxide dismutase (SOD) and ascorbate peroxidase (APX) activities in conjunction with the catalase activity [16], and enhancing the gene expression of chlorophyll and rubisco-binding proteins [19]. Recent studies have demonstrated that ZnO, Fe_2_O_3_, TiO_2,_ and CeO_2_ NPs can alleviate abiotic stress in plant, but not enough about these NPs on chilling stress is currently understood. Accordingly, we supposed that NPs’ application could relieve the negative effects of chilling stress on direct-seeded early indica rice seedlings. In order to test our hypothesis, we conducted a pot experiment to study the impact of chilling stress on agronomic traits, osmolyte, and antioxidant enzymes as well the chlorophyll content in early indica rice at the seedling stage. In this study, the main objective of our experiment is to elucidate the physiological traits and growth differences of cultivars under cold stress at the seedling stage and, moreover, further elaborate on some mechanism governing nanoparticles as well temperature in direct-seeded early indica rice during its seedling phase. Therefore, NPs were sprayed to investigate whether they could alleviate chilling stress damage in the current research.

## 2. Results

### 2.1. XRD Analysis of Nanoparticles

**Figure 1:**
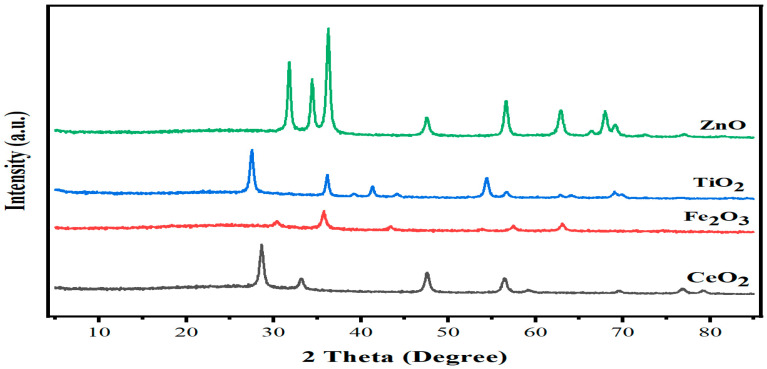
X-ray diffraction (XRD) pattern of the applied nano-particles used in this experiments.

The X-ray diffraction (XRD) analysis reveals the single-phase, crystalline structure of the ZnO sample. The XRD analysis of the ZnO sample was carried out from a 10 to 80 Bragg’s angle with a step size 0.2 s, and the scan speed was taken as 10 per minute. Figure 1 shows the XRD pattern of ZnO which was analyzed by the Origin 2023 software. The XRD analysis of the ZnO sample represents the single-phase crystalline structure having high-intensity peaks which indicate the crystalline structure of the powder sample. The typical XRD spectrum of titanium oxide is represented in Figure 1, where the spectrum shows 2θ values from 26.3, 35.8, 41.7, 42.089, 54.9, 55.6, 64.28, 65.7, and 68.9. The phase composition and the crystal structure of iron oxide studied by an X-ray diffractometer are represented in Figure 1. In the XRD diffractogram, the characteristic peaks of the samples are at 2θ = 29.7°, 35.50°, 43.25°, 57.56°, 57.14°, and 64.73°. The typical XRD spectrum of cerium oxide is as depicted in Figure 1, where the spectrum shows 2θ values from 28.302, 32.851, 47.270, 56.089, 58.889, 69.178, 76.528, and 78.838, which corresponds to hkl planes at 111, 200, 220, 311, 222, 400, 331, and 420, respectively.

### 2.2. Effect of Nanoparticles on Plant Growth Parameters

Zinc oxide nanoparticles significantly influenced the plant height, fresh weight, dry weight, and root length compared to the control (CK). The increase in the plant height was 22.8% at 100 mg/L ZnO compared to CK, while the untreated plants (0 mg/L ZnO) showed a decrease of 45.7%. The fresh weight increased by 25.5% at 100 mg/L ZnO compared to CK, whereas the untreated plants exhibited a 44.2% decrease. The dry weight shows an increase of 22.9% at 100 mg/L ZnO compared to CK, with the untreated plants showing a 46.9% decrease. The root length increased by 16.3% at 100 mg/L ZnO compared to 0 mg/L, while the untreated plants exhibited a decrease of 47.9%. The LSD values for the plant height, fresh weight, dry weight, and root length were 10.2%, 9.8%, 7.3%, and 11.8%, respectively (Table 1). Iron oxide nanoparticles also significantly affected plant growth. The highest increase in the plant height (18.2%) was observed at 50 mg/L Fe_2_O_3_ compared to CK, whereas untreated plants (0 mg/L Fe_2_O_3_ i.e., water spray) showed a 50.1% decrease. The fresh weight increased by 17.9% at 50 mg/L FeO compared to CK, with untreated plants showing a 45.5% decrease. The dry weight increased by 16.2% at 50 mg/L Fe_2_O_3_ compared to CK, while untreated plants exhibited a 47.7% decrease. The root length increased by 20.6% at 50 mg/L Fe_2_O_3_ compared to CK, with untreated plants showing a 45.6% decrease. The LSD values for plant height, fresh weight, dry weight, and root length were 8.3%, 11.4%, 8.2%, and 11.9%, respectively (Table 1). The application of titanium oxide nanoparticles resulted in significant variations in the plant growth parameters. At 50 mg/L TiO_2_, the plant height increased by 22.2% compared to CK, while the untreated plants (0 mg/L TiO_2_) showed a 52.7% decrease. The fresh weight increased by 15.7% at 50 mg/L TiO_2_ compared to CK, with untreated plants showing a 43.3% decrease. The dry weight increased by 15.7% at 50 mg/L TiO_2_ compared to CK, while the untreated plants exhibited a 47.3% decrease. The root length increased by 19.0% at 50 mg/L TiO_2_ compared to CK, with the untreated plants showing a 45.5% decrease. The LSD values for plant height, fresh weight, dry weight, and root length were 8.1%, 7.4%, 6.9%, and 8.8%, respectively (Table 1). Cerium oxide nanoparticles had a notable impact on the growth parameters of the plants. The highest increase in plant height (17.8%) was obtained at 75 mg/L CeO_2_ compared to CK, while the untreated plants (0 mg/L CeO_2_) showed a 42.5% decrease. The fresh weight increased by 22.7% at 75 mg/L CeO_2_ compared to CK, with the untreated plants showing a 38.2% decrease. The dry weight increased by 24.8% at 75 mg/L CeO_2_ compared to CK, while the untreated plants exhibited a 53.5% decrease. The root length increased by 24.3% at 75 mg/L CeO_2_ compared to CK, with the untreated plants showing a 46.3% decrease. The LSD values for plant height, fresh weight, dry weight, and root length were 9.7%, 8.5%, 8.6%, and 14.1%, respectively (Table 1). Among the cultivars tested, LLY-7108 showed the highest values for plant height (27.8 cm), fresh weight (16.45 g/plant), dry weight (1.159 g/plant), and root length (15.94 cm), with increases of 36.9%, 39.1%, 32.5%, and 45.1% compared to the lowest values observed in Zhang-17, respectively. Zhang-17 recorded the lowest values with decreases of 27.0%, 27.8%, 24.5%, and 31.1% for plant height, fresh weight, dry weight, and root length compared to the highest values in LLY-7108. The LSD values for plant height, fresh weight, dry weight, and root length were 10.1%, 7.5%, 7.5%, and 11.5%, respectively (Table 1).

### 2.3. Effect of Nanoparticles on Photosynthetic Pigment

The effect of zinc oxide nanoparticles and chilling stress on the chlorophyll A content was significant across all tested cultivars. In LLY-7108, the chlorophyll A content at an ambient temperature was the highest at approximately 250 µg/g FW. Under 0 mg/L ZnO + chilling stress, the chlorophyll A content decreased by 30%, dropping to around 175 µg/g FW (Table 1). Treatments with 50 mg/L and 100 mg/L ZnO + chilling stress mitigated this reduction, resulting in chlorophyll A contents of about 210 µg/g FW (16% decrease) and 225 µg/g FW (10% decrease). Similarly, chlorophyll B is significantly influenced by zinc oxide nanoparticles and chilling stress. The chlorophyll A content was significant across all tested cultivars. In LLY-7108, the chlorophyll A content at an ambient temperature was the highest at approximately 250 µg/g FW. Under 0 mg/L ZnO + chilling stress, the chlorophyll A content decreased by 30%, dropping to around 175 µg/g FW (Table 1). Treatments with 50 mg/L and 100 mg/L ZnO + chilling stress mitigated this reduction, resulting in chlorophyll A contents of about 210 µg/g FW (16% decrease) and 225 µg/g FW (10% decrease). Iron oxide nanoparticles significantly affected the photosynthetic pigment. The decrease in chlorophyll A under the chilling stress condition was 27.97% compared to the ambient temperature (Table 1). The chlorophyll B content at an ambient temperature was the highest across all the cultivars; under 0 mg/L Fe_2_O_3_ + chilling stress, the chlorophyll B content decreased by 32.48%, dropping to around 121.45 µg/g FW (Table 1) across all the cultivars. Chlorophyll B increased by 24.06% at 50 mg/L Fe_2_O_3_ compared to 0 mg/L + chilling stress. The foliar application of titanium oxide nanoparticles resulted in significant variations in the plant photosynthetic pigment. At 50 mg/L TiO_2_, chlorophyll A increased across all the cultivars by 29.76% compared to untreated plants (0 mg/L TiO_2_), showing a 68.8% decrease as compared to the ambient temperature (Table 1). Rice plants under an ambient temperature resulted with the highest chlorophyll B content (180.1 µg/g FW) across all the cultivars. The increase in the chlorophyll B content was 27.5%, 16.9%, and 9.02% at 50, 75, and 100 mg/L TiO_2_, respectively, compared to 0 mg/L + chilling stress (Table 1). Cerium oxide nanoparticles had a notable impact on the plant photosynthetic pigment. The highest increase in chlorophyll A (31.04%) was obtained at 75 mg/L CeO_2_ compared to the untreated plants (0 mg/L CeO_2_), which showed a 51.08% decrease compared to the ambient temperature (Table 1). The chlorophyll B content was found to be the highest (185.2 µg/g FW) for the ambient temperature. The increase in the chlorophyll B content was by 27.8% at 75 mg/L CeO_2_ compared to the untreated plants, which exhibited a 51.25% decrease (Table 1). Among the cultivars tested, LLY-7108 showed the highest values for Chlorophyll A and B (180.78 ug/g FW and 163.93 ug/g FW), with increases of 24.1% and 21.66% compared to the lowest values observed in ZJZ-17.

### 2.4. Application of ZnO, FeO, TiO, and CeO_2_ NPs Alleviate Chilling-Induced Oxidative Stress in Rice

**Figure 2:**
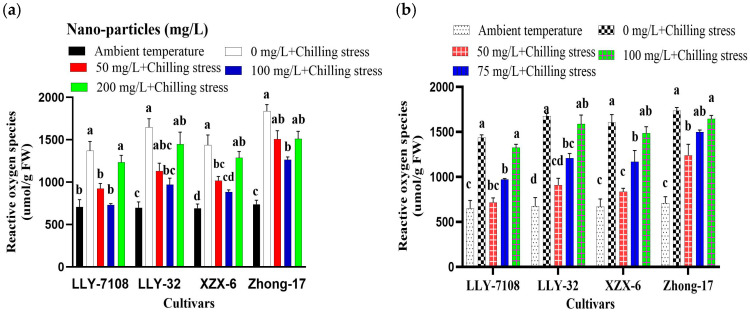

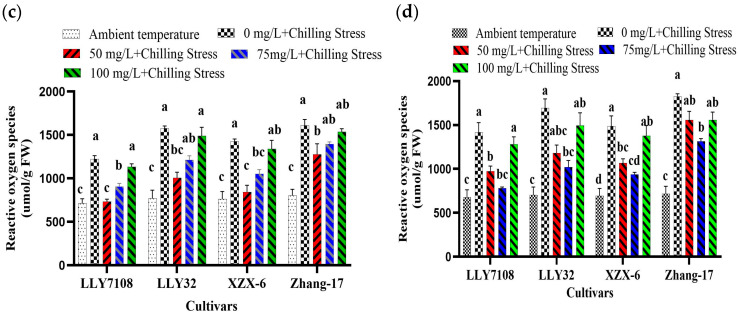
Reactive oxygen species of rice seedlings subjected to ZnO (**a**), Fe_2_O_3_ (**b**), TiO_2_ (**c**), and CeO_2_ (**d**) NPs under chilling stress. Data are the means of three replicates ± SD (error bars). Means with the same lower-case letters are not significantly different among varieties; differences between treatments means were tested with Tukey’s test at 5% probability level.

**Figure 3:**
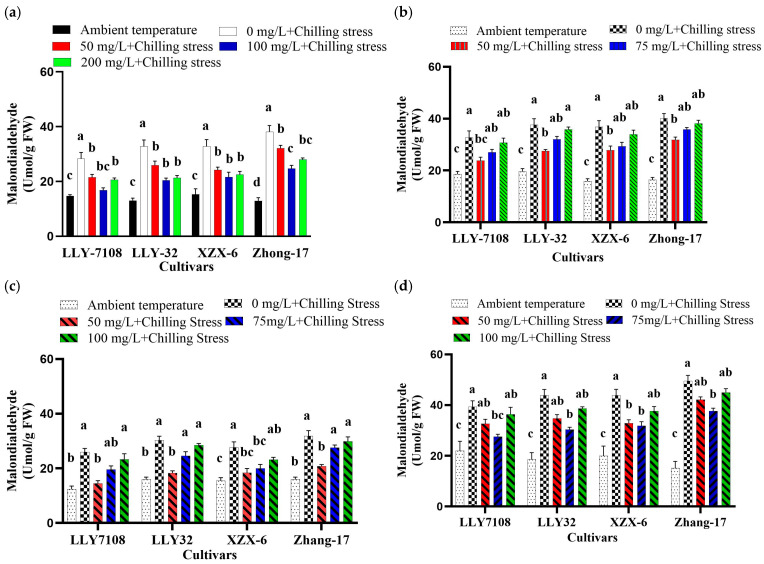
Malonaldehyde of rice seedlings subjected to ZnO (**a**), Fe_2_O_3_ (**b**), TiO_2_ (**c**), and CeO_2_ (**d**) NPs under chilling stress. Data are the means of three replicates ± SD (error bars). Means with the same lower-case letters are not significantly different among varieties; differences between treatments means were tested with Tukey’s test at 5% probability level.

**Figure 4:**
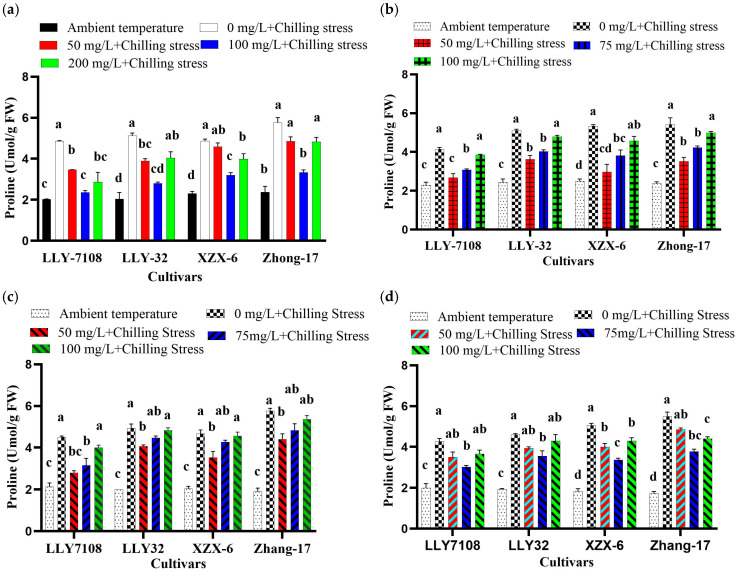
Proline of rice seedlings subjected to ZnO (**a**), Fe_2_O_3_ (**b**), TiO_2_ (**c**), and CeO_2_ (**d**) NPs under chilling stress. Data are the means of three replicates ± SD (error bars). Means with the same lower-case letters are not significantly different among varieties; differences between treatments means were tested with Tukey’s test at 5% probability level.

To evaluate the effect of ZnO, Fe_2_O_2_, TiO_2_, and CeO_2_ NPs’ application on chilling stress, 21-day-old rice plants were exposed to the chilling condition (12 h light/12 h dark, at 14 °C/10 °C). Low temperatures (14/10 °C) induced phytotoxic retards in the rice seedlings. Chilling stress led to the increasing of the ROS contents, which indicated that the chilling processes produced serious oxidative stresses, which is extremely harmful for plant cells. The rice leaves treated under chilling stress generated 1.22-, 1.35-, and 1.34-fold higher levels of ROS, MDA, and proline than the control (Figure 2, Figure 3 and Figure 4). The supplementation of 100 mg/L ZnO NPs significantly reduced the ROS, MDA, and proline contents in rice leaves by 38.75% (Figure 2a), 36.66% (Figure 3a), and 43.60% (Figure 4a), respectively, compared to chilling stress. Iron oxide nanoparticles significantly reduced the oxidative stress in rice. The decrease in ROS, MDA, and proline in rice leaves under the chilling stress condition is observed under iron supplementation. The ROS, MDA, and proline content at an ambient temperature was the lowest across all the cultivars; under 0 mg/L Fe_2_O_3_ + chilling stress, the ROS, MDA, and proline contents increased. The ROS, MDA, and proline content in rice leaves reduced by 42.65% (Figure 2b), 24.93% (Figure 3b), and 36% (Figure 4b) at 50 mg/L Fe_2_O_3_ compared to 0 mg/L + chilling stress. The foliar application of titanium oxide nanoparticles resulted in significant variations in ROS, MDA, and proline. At 50 mg/L TiO_2_, ROS, MDA, and proline decreased by 33.67% (Figure 2c), 37.7% (Figure 3c), and 25.7% (Figure 4c), respectively, across all of the cultivars compared to the untreated plants (0 mg/L TiO_2_). Cerium oxide nanoparticles had a notable impact on the plant ROS, MDA, and proline. The highest increase in ROS, MDA, and proline was recorded at 0 mg/L CeO_2_ compared to all the other treatments (Figure 2d, Figure 3d and Figure 4d). The ROS, MDA, and proline content was found to be increased under chilling stress by 1.31-, 1.32-, and 1.61-folds, respectively. The reduction in ROS, MDA, and proline was by 37.56% (Figure 2d), 27.9% (Figure 3d), and 29.4% (Figure 4d) at 75 mg/L CeO_2_ compared to untreated plants under chilling stress. Among the cultivars tested, LLY-7108 showed resistance to chilling stress and had the minimum oxidative stress. The highest value of ROS (1090.7), MDA (25.9), and proline (3.5) was recorded for Zhang-17 across all the NPs’ applications compared to other cultivars.

### 2.5. Impact of NPs on Antioxidant Enzymes

**Figure 5:**
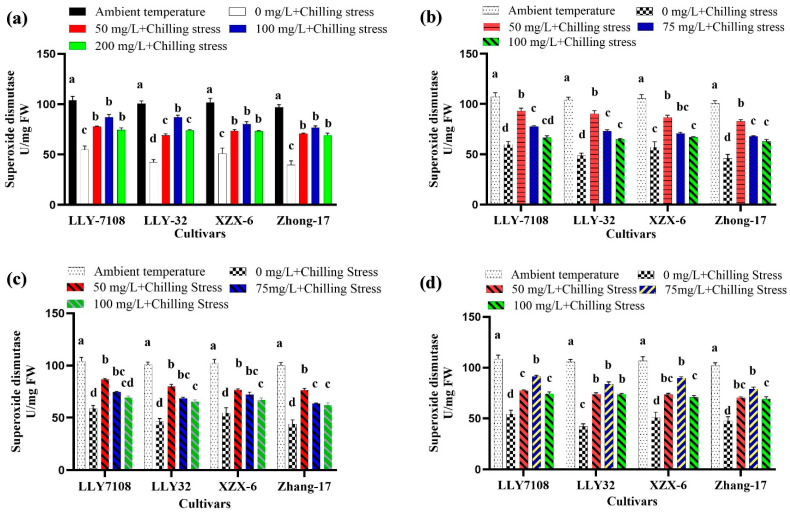
Superoxide dismutase of rice seedlings subjected to ZnO (**a**), Fe_2_O_3_ (**b**), TiO_2_ (**c**), and CeO_2_ (**d**) NPs under chilling stress. Data are the means of three replicates ± SD (error bars). Means with the same lower-case letter are not significantly different among varieties; differences between treatments means were tested with Tukey’s test at 5% probability level.

**Figure 6:**
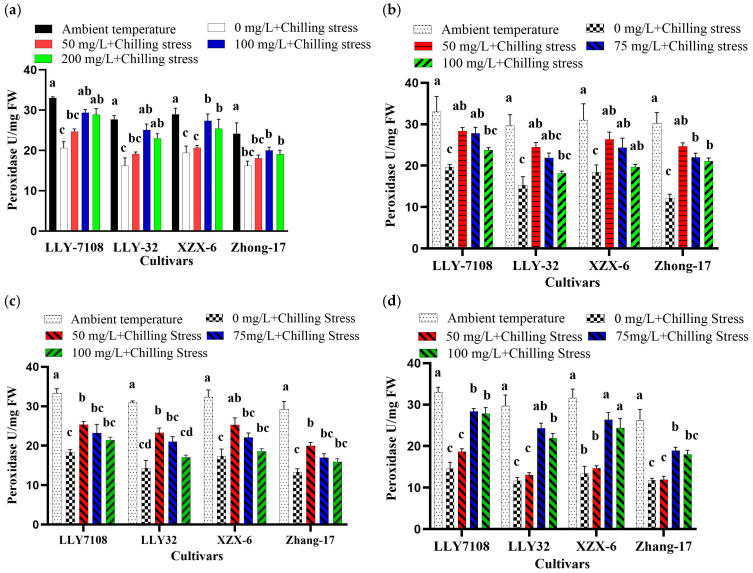
Peroxidase of rice seedlings subjected to ZnO (**a**), Fe_2_O_3_ (**b**), TiO_2_ (**c**), and CeO_2_ (**d**) NPs under chilling stress. Data are the means of three replicates ± SD (error bars). Means with the same lower-case letter are not significantly different among varieties; differences between treatments means were tested with Tukey’s test at 5% probability level.

**Figure 7:**
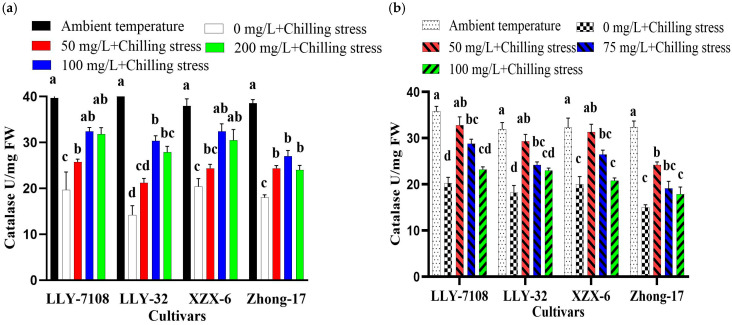

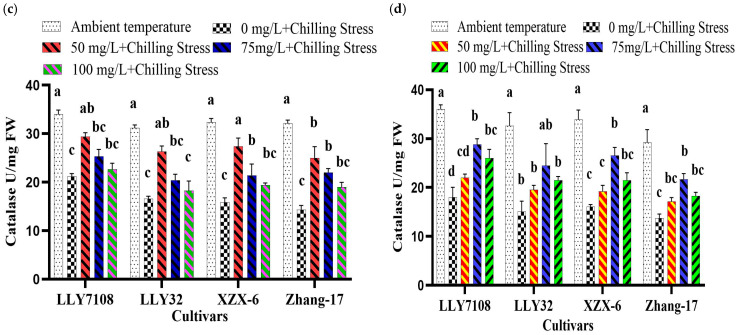
Catalase of rice seedlings subjected to ZnO (**a**), Fe_2_O_3_ (**b**), TiO_2_ (**c**), and CeO_2_ (**d**) NPs under chilling stress. Data are the means of three replicates ± SD (error bars). Means with the same lower-case letter are not significantly different among varieties; differences between treatments means were tested with Tukey’s test at 5% probability level.

Nanoparticles mitigated Chilling-Induced Oxidative Stress in rice. To gain further insight regarding the alleviation of chilling stress by ZnO NPs, we also determined SOD, CAT, and POD activities in rice plants leaves. Low-temperature stress significantly reduced the antioxidant enzyme (SOD, CAT, and POD) activity. Nevertheless, the induced SOD, CAT, and POD activities in rice seedlings were considerably increased when subjected to NPs compare to chilling stress alone. However, the application of NPs greatly increased the activities of SOD, CAT, and POD in chilling-treated rice seedlings compared to those under only chilling stress. Chilling stress treatment strongly reduced the activity of SOD, POD, and CAT in rice leaves by 107%, 91.345%, and 98.6%, respectively, compared to the control under chilling stress (Figure 5, Figure 6 and Figure 7). The foliar application of 100 mg/L ZnO NPs significantly increased the activity of SOD, POD, and CAT in rice leaves by 76.08% (Figure 5a), 40.1% (Figure 6a), and 68.87% (Figure 7a), respectively, compared to chilling stress. Iron oxide nanoparticles significantly altered the oxidative stress in rice. The increase in the SOD, POD, and CAT activities in rice leaves under the chilling stress condition was observed with iron supplementation. The SOD, POD, and CAT activity at an ambient temperature was the highest across all the cultivars; under 0 mg/L Fe_2_O_3_ + chilling stress, the SOD, POD, and CAT activity reduced. The antioxidant enzymatic activity (SOD, POD, and CAT) in rice leaves increased by 67% (Figure 5b), 58.1% (Figure 6b), and 59.93% (Figure 7b) at 50 mg/L Fe_2_O_3_ compared to 0 mg/L + cold stress. The foliar application of titanium oxide nanoparticles resulted in significant variations in the SOD, POD, and CAT activity. At 50 mg/L TiO, the SOD, POD, and CAT activity decreased by 56.69% (Figure 5c), 46.65% (Figure 6c), 25.7%, and 59.04% (Figure 7c), respectively, across all the cultivars compared to the untreated plants (0 mg/L TiO_2_). Cerium oxide nanoparticles had a notable impact on the plant’s SOD, POD, and CAT activity. The highest increase in the SOD, POD, and CAT activity was recorded at 0 mg/L CeO_2_ compared to all other treatment (Figure 5d, Figure 6d and Figure 7d). The reduction in the SOD, POD, and CAT activity was by 76.4% (Figure 5d), 90.6% (Figure 6d), and 61.18% (Figure 7d) at 75 mg/L CeO_2_ compared to the untreated plants under chilling stress. Among the cultivars tested, LLY-7108 showed resistance to chilling stress and had the minimum antioxidant enzymatic activity. The activity of the antioxidant enzyme was lowest in Zhang-17 across all the NP applications compared to the other cultivars.

### 2.6. Principal Component, Correlation Plots, and Regression Analysis

**Figure 8:**
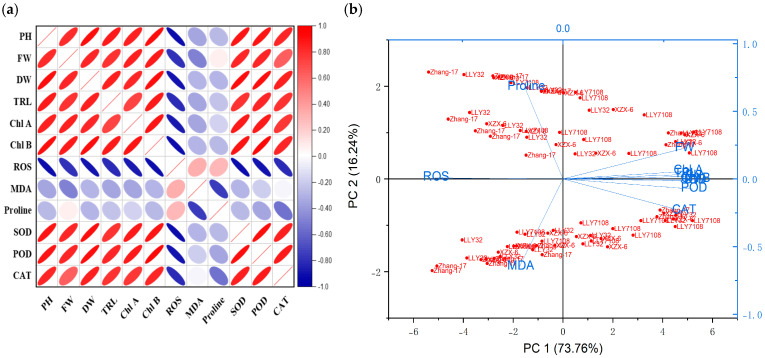
Correlation analysis of the studied traits of rice seedlings influence by chilling stress and nano-particle applications (**a**). Bi-plot (P.C.A.) presenting the correlation between the tested traits of rice seedlings as influenced by chilling stress and nano-particle applications (**b**).

**Figure 9:**
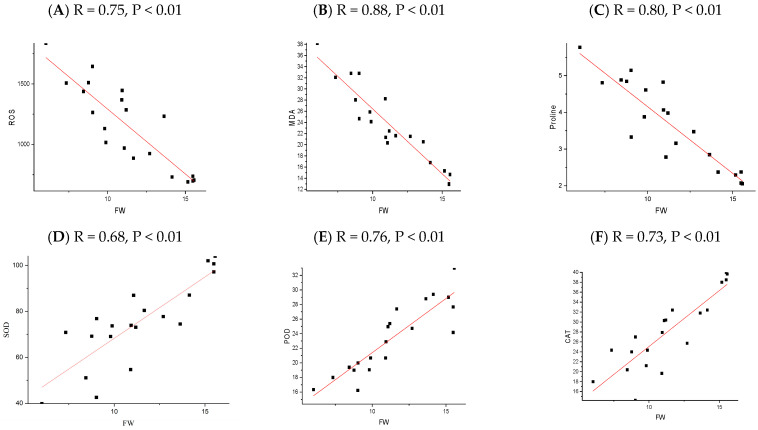
The relationship between rice physiological parameter (ROS (**A**), MDA (**B**), Proline (**C**), SOD (**D**), POD (**E**), and CAT (**F**)) and fresh weight, as influenced by chilling stress and ZnO nanoparticles application. The red line refers to the regression line or line of best fit, which is a straight line that best represents the relationship between the physiological parameter and the fresh weight of rice seedlings. The black square represents the actual data point.

**Figure 10:**
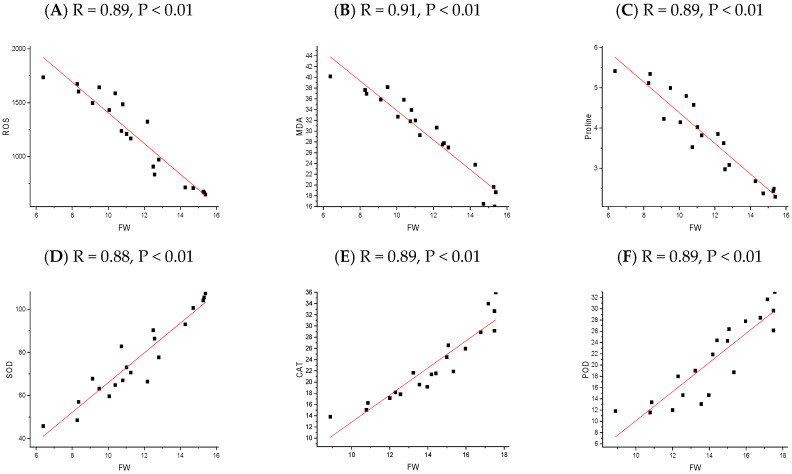
The relationship between rice physiological parameters (ROS (**A**), MDA (**B**), Proline (**C**), SOD (**D**), POD (**E**), and CAT (**F**)) and fresh weight, as influenced by chilling stress and Fe_2_O_3_ nanoparticles application. The red line refers to the regression line or line of best fit, which is a straight line that best represents the relationship between the physiological parameter and the fresh weight of rice seedlings. The black square represents the actual data point.

**Figure 11:**
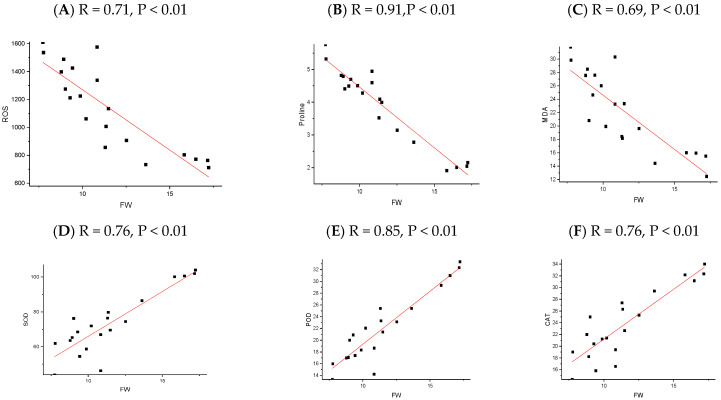
The relationship between rice physiological parameters (ROS (**A**), MDA (**B**), Proline (**C**), SOD (**D**), POD (**E**), and CAT (**F**)) and fresh weight, as influenced by chilling stress and TiO_2_ nanoparticles’ application. The red line refers to the regression line or line of best fit, which is a straight line that best represents the relationship between the physiological parameter and the fresh weight of rice seedlings. The black square represents the actual data point.

**Figure 12:**
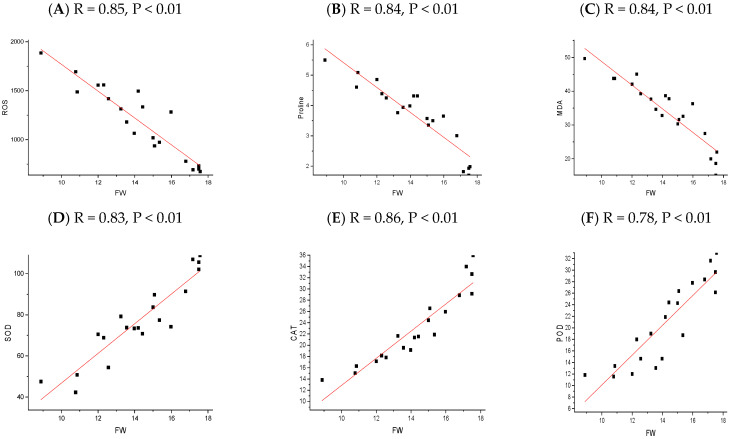
The relationship between rice physiological parameters (ROS (**A**), MDA (**B**), Proline (**C**), SOD (**D**), POD (**E**), and CAT (**F**)) and fresh weight, as influenced by chilling stress and CeO_2_ nanoparticles application. The red line refers to the regression line or line of best fit, which is a straight line that best represents the relationship between the physiological parameter and the fresh weight of rice seedlings. The black square represents the actual data point.

The correlation analysis revealed significant and robust correlations, notably between the plant height (PH), fresh weight (FW), dry weight (DW), root length (RL), SOD, POD, CAT, chlorophyll A, and chlorophyll B. In contrast, MDA, ROS, and Proline were negatively correlated across all the NP applications. A P.C.A. analysis was performed using a biplot to investigate the influence NPs and different cultivars under chilling stress (Figure 8). The principal component analysis (P.C.A.) showed that different traits were strongly correlated, and various treatments were differentially distributed (Figure 8b). The biplot revealed that the studied traits accounted for 73.76% of the total variability of the data. The first P.C. revealed 73.76% of the total variation, while PC2 accounted for 16.24%. The biplot revealed that PC1 was correlated with almost all of the studied traits, which was positively associated. There was a significant negative correlation between the osmolytes and fresh weight of the rice seedling influenced by chilling stress (Figure 9, Figure 10, Figure 11 and Figure 12); however, the relationship between the antioxidant enzyme activity and fresh weight was found to be positive (Figure 9, Figure 10, Figure 11 and Figure 12).

## 3. Materials and Methods

### 3.1. Plant Materials and Growth Conditions

In present study, four early indica rice cultivars, Zhongjiazao17 (ZJZ-17), Xiangzaoxian6 (XZX-6), Lingliang you-7108 (LLY-7108), and Lingliang you-32 (LLY-32), were selected for pot experiments. ZJZ-17 is a chilling sensitive cultivar which is bred by the China National Rice Research Institute (CNRRI). XZX-6 is a chilling-tolerant cultivar bred by the Hunan Yuanjiang Agricultural Research Institute, (CNRRI) [20]. LLY-7108, a chilling-tolerant cultivar, was bred by Hunan Institute of Yahua Seed Industry Research Institute. LLY-32, a chilling-sensitive cultivar, was bred by Hunan Institute of Yahua Seed Industry Research Institute, China Institute of Rice Research. The pot experiment was carried out in Huazhong Agricultural University 114.37° E, 30.48° N, Wuhan, China. The pot experiment had pots which were 15.0 cm in height, 25.0 cm in length, and 23.0 cm in width; 6.0 kg soil was used in each pot and 0.96 g nitrogen, 0.92 g phosphorus, and 112 g potassium/pot each was applied. Fertilizers were mixed into the soil 2 day before the pot direct seeding. The soil sample was collected from the topsoil layer at a 0–20 cm soil depth in the rice paddy experiment field. The soil physical and chemical properties were pH 7.1, organic matter 6.7 g/kg, Olsen-P 6.27 mg/kg, exchangeable K 129 mg/kg, and total N 0.63%.

### 3.2. Experimental Design and Treatments

Rice (*Oryza sativa*) seeds were sterilized with 20% (*v*/*v*) NaClO and soaked into ultra-pure water at 30 °C for two days. A pot consisted of 10 rice seeds and was cultured under normal conditions in a growth chamber with relative humidity of 60% (12 h light at 30 °C/12 h dark at 25 °C). Foliage sprays of different NPs, ZnO (0, 50, 100, and 200 mg/L), Fe_2_O_3_ (0, 50, 75, and 100 mg/L), Tio_2_ (0, 50, 75, and 100 mg/L), and CeO_2_ (0, 50, 75, and 100 mg/L), were applied to rice plant as a foliage spray (15 mL plant^−1^). For chilling stress treatment, 21-day-old rice plants were subjected to low-temperature stress condition (12 h light 14 °C/12 h dark, at 10 °C) for five days. Next, the chilling-treated rice seedlings were transferred to a normal condition for 1 week. The rice seedlings, after 32 days of functioning with or without NPs treatment, were sampled for phenotypic and physiological characterization. Three plants per treatment were measured (three replicates) for plant height, roots length, and dry biomasses, as well 3–6 plants/replicate depending on the weight to be evaluated in other different physiological measurements and biochemical detection.

### 3.3. Details of NPs

The NPs of ZnO, Fe_2_O_3_, TiO_2_, and CeO_2_ were purchased from Shanghai Aladdin Bio-Chem Technology Co., Ltd., Shanghai, China. The particle morphology and size distribution of ZnO, Fe_2_O_3_, TiO_2_, and CeO_2_ NPs were observed by X-ray diffraction. Powder X-ray diffraction (XRD) data were recorded and collected on the XRD model MPD X’PERT PRO of PANalytical Co., Ltd., Malvern, UK, Holland, using Cu–Ka as characteristic radiation (k = 0.15418 nm) with h–h configuration. The measurements were made in 2 h ranging from 20 to 80. To minimize agglomeration, we sonicated the different concentrations of ZnO, Fe_2_O_3_, TiO_2_, and CeO_2_ NPs’ suspensions for 1 h before use.

### 3.4. Agronomic Characters

Three seedlings per treatment were selected, and each treatment was repeated three times. The plant height, root length, fresh weight, and dry weight were calculated after washing and drying the seedlings. The dry weight of the aboveground parts was determined after oven-drying at 80 °C to achieve a constant weight.

### 3.5. Determination of Chlorophyll (Chl.)

The concentration of chlorophyll in rice leaves was measured using the method described by Arnon [21]. A total of 0.5 g of freshly harvested rice leaves were thoroughly combined and pulverized with 10 mL of 80% (*v*/*v*) acetone. Following a 2 h period of light deprivation, the samples were then centrifuged at 12,000 rpm for 10 min. Ultimately, the absorbance was measured at wavelengths of 663 and 645 nm, respectively, with spectrophotometer.

### 3.6. Measurement of Malondialdehyde (MDA) Content

A previously described method of Heath and Packer was used for the quantification of MDA [22]. For the grinding of plant material, an approximate amount of 0.5 g fresh weight leaves were homogenized with 10 mL ice cold solution, consisting of 1/5 M trichloroacetic acid. The resulting mixture was then centrifuged at 12,000× *g* for 20 min. Then, 2 mL of the supernatant was mixed with equal volume of thiobarbituric acid and incubated at 95 °C for 30 min. Then the mixture was centrifuged under a force of 12,000× *g* for thirty minutes following rapid cooling in ice, and the supernatant absorbance at 450, 532, and 600 nm was measured, respectively, with spectrophotometer.

### 3.7. Measurement of Free Proline (Pro)

The measurement of free proline content was conducted following the methodology described in a previous study by He et al. [9]. Approximately 1 g of rice leaves were ground using a solution of sulfosalicylic acid at a concentration of 3% (*v*/*v*) in water. After centrifugation at room temperature at 12,000× *g* for 15 min, 2 mL of supernatant was added to the same glacial acetic acid and acidic ninhydrin volumes. The mixture was subjected to heating at a temperature of 95 °C for a duration of 40 min. Subsequently, it was cooled using ice, then 4 mL of toluene was added to extract. The absorbance was measured at a wavelength of 520 nm with spectrophotometer, and the standard curve of proline was used to analyze the concentration of accumulated free proline.

### 3.8. Activity of Antioxidant Enzyme

To determine antioxidants activities, fresh leaf samples (0.1 g) were ground in 1 mL of cold phosphate buffer (50 mM; pH-7.8). After homogenate solution were centrifuged at 12,000× *g* for 20 min at the condition of 4 °C. The activity superoxide dismutase (SOD, EC1.15.1) was measured according to [23]. Peroxidase (POD, EC.1.1.1) was measured at absorbance 470 nm using the method of Thipyapong et al. [24]. Activity of catalase (CAT, EC 1.11.1.6) was assayed according to [25] with spectrophotometer.

### 3.9. Statistical Analysis

Data were analyzed using a two-way analysis of variance (SAS Institute, Inc., Cary, NC, USA), and differences between treatments’ means were tested with Tukey’s test at 5% probability level.

## 4. Discussion

The research has demonstrated that the growth of rice seedlings is significantly hindered when the mean temperature falls below 12 °C. Applications of ZnO NPs increase resistance to stresses (drought stress, salinity stress, and cadmium and lead toxicity), which has been reported in several plants. Under drought stress and salt stress, the addition of ZnO, Fe_2_O_3_, TiO_2_, and CeO_2_ nanoparticles (NPs) enhanced the growth and biochemical and physiological parameters. Nevertheless, there is a lack of research that has examined the impact of ZnO, Fe_2_O_3_, TiO_2_, and CeO_2_ NPs on rice seedlings subjected to chilling stress. This study demonstrates that stress has a detrimental effect on the height, fresh weight, and dry weight of rice seedlings. The growth of rice seedlings in terms of their height, fresh weight, and dry weight showed a significantly higher increase when NPs were applied compared to experiencing only a water spray under cold stress. This is due to the limited ability of rice seedlings to absorb nutrients and reducing their assimilated production could be a key factor contributing to their slow recovery in terms of their fresh and dry weight [26,27]. Chilling stress has a detrimental impact on the metabolic and physiological functions of rice, leading to a decrease in yield by impeding the formation of chloroplasts and the biosynthesis of chlorophyll in leaves [28,29]. Consistent with previous studies, the rice seedlings in this study displayed visible chlorosis in young leaves, accompanied by a significant decrease in the overall content of chlorophyll (both chlorophyll A and B) after exposure to chilling stress, compared to the untreated control [30,31]. However, the application of ZnO nanoparticles (NPs) alleviated these symptoms and effectively restored the accumulation of chlorophyll A and chlorophyll B (Table 1). This suggests that ZnO NPs can mitigate the adverse effects of chilling stress on photosynthetic pigments by enhancing chlorophyll synthesis or safeguarding the structure of chloroplasts. Similarly, Fe_2_O_3_ NPs showed a protective effect on the chlorophyll B content under chilling stress. Without Fe_2_O_3_ treatment, the chlorophyll B content decreased by 32.48%, but with 50 mg/L Fe_2_O_3_, there was a 24.06% increase compared to untreated stressed plants. This suggests that FeO NPs may enhance chlorophyll stability or synthesis, which is crucial in maintaining photosynthetic efficiency under stress conditions [32,33]. TiO_2_ and CeO_2_ nanoparticles also had a significant impact on photosynthetic pigments. TiO_2_ NPs at 50 mg/L increased their chlorophyll A content by 29.76% (Table 1), and CeO_2_ NPs at 75 mg/L boosted their chlorophyll A content by 31.04% under cold stress (Table 1). These findings are consistent with the potential of these nanoparticles to enhance the plant’s photosynthetic capacity and stress resilience by various mechanisms, such as improving light absorption or protecting chloroplasts from oxidative damage [34,35].

Reactive oxygen species (ROS) have the ability to incorporate environmental stress signals, activate gene expression as a reaction to stress, and thus establish an equilibrium between plant defense and growth [36]. Stress triggers the overproduction of reactive oxygen species (ROS), such as O^−2^, H_2_O_2_, and OH, in plants [37]. This leads to oxidation and higher concentrations of soluble proteins, which can enhance the plant’s ability to withstand stress. However, it can also affect normal physiological processes in plants [38,39]. Antioxidant enzymes, such as superoxide dismutase (SOD), peroxidase (POD), and catalase (CAT), have the ability to remove excessive reactive oxygen species (ROS). Exposure to chilling stress triggers the release of excessive reactive oxygen species (ROS) and malondialdehyde (MDA), leading to oxidative damage and stress. Additionally, there is an increase in the proline levels, indicating the presence of oxidative damage and stress. This study revealed that the application of ZnO NPs at a concentration of 100 mg/L resulted in a substantial decrease of 38.75%, 36.66%, and 43.60% in the ROS, MDA, and proline contents, respectively, compared to stressed plants that were not treated with ZnO NPs (Figure 2a, Figure 3a and Figure 4a). This reduction demonstrates the role of ZnO NPs in enhancing the antioxidative defense mechanisms, possibly through the activation of antioxidant pathways or the direct scavenging of ROS. Fe_2_O_3_ NPs at 50 mg/L also reduced oxidative stress markers, with ROS, MDA, and proline contents decreased by 42.65%, 24.93%, and 36%, respectively (Figure 2b, Figure 3b and Figure 4b). This suggests that Fe_2_O_3_ NPs enhance the plant’s antioxidative defenses by providing essential iron for the functioning of antioxidative enzymes and metabolic processes [40]. The effects of TiO_2_ and CeO_2_ nanoparticles were also significant. At 50 mg/L, TiO_2_ NPs reduced the ROS, MDA, and proline levels by 33.67%, 37.7%, and 25.7%, respectively (Figure 2c, Figure 3c and Figure 4c). Similarly, CeO_2_ NPs at 75 mg/L reduced these oxidative stress markers by 37.56%, 27.9%, and 29.4%, respectively (Figure 2d, Figure 3d and Figure 4d). These findings showed the potential of these nanoparticles to mitigate oxidative stress by enhancing antioxidative enzyme activities or other protective mechanisms.

This study also highlighted that ZnO, Fe_2_O_3_, TiO_2_, and CeO_2_ nanoparticles significantly enhance the activities of key antioxidant enzymes such as superoxide dismutase (SOD), catalase (CAT), and peroxidase (POD) in rice plants under chilling stress. ZnO NPs at 100 mg/L increased the activities of SOD, POD, and CAT by 76.08%, 40.1%, and 68.87%, respectively, compared to untreated stressed plants (Figure 5a, Figure 6a and Figure 7a). This suggests that ZnO NPs activate the plant’s antioxidative defense system, crucial for detoxifying ROS and protecting cells from oxidative damage [41]. Fe_2_O_3_ NPs at 50 mg/L similarly increased SOD, POD, and CAT activities by 67%, 58.1%, and 59.93%, respectively, under chilling stress (Figure 5b, Figure 6b and Figure 7b). The increase in the antioxidant enzyme activities indicates that Fe_2_O_3_ NPs facilitate better iron nutrition, essential for the optimal functioning of these enzymes [42]. TiO_2_ and CeO_2_ nanoparticles also had positive effects on the antioxidant enzyme activities. At 50 mg/L, TiO_2_ NPs increased SOD, POD, and CAT activities by 56.69%, 46.65%, and 59.04%, respectively (Figure 5c, Figure 6c and Figure 7c). CeO_2_ NPs at 75 mg/L also enhanced these enzyme activities, highlighting their role in boosting the antioxidative defense system of plants under chilling stress. Chilling stress also affects the expression of various transcription factors involved in the chilling tolerance and response in rice. However, the application of ZnO NPs reduced the expression of these chilling-stress-induced genes, thereby alleviating the toxic effects of chilling stress. This suggests that ZnO NPs help modulate gene expression, contributing to the plant’s stress tolerance mechanisms.

## 5. Conclusions and Future Perspectives

This study’s results indicate that applying ZnO, Fe_2_O_3_, TiO_2_, and CeO_2_ nanoparticles to the leaves of rice plants can successfully alleviate the negative impacts of chilling stress. The nanoparticles improve the plant’s ability to retain photosynthetic pigments, decrease signs of oxidative stress, and enhance the activities of antioxidant enzymes, thereby enhancing the plant’s overall resilience to stress. In conclusion, we demonstrated that the foliar application of ZnO (100 mg/L), Fe_2_O_3_ (50 mg/L), TiO_2_ (50 mg/L), and CeO_2_ (75 mg/L) NPs could efficiently mitigate the toxicity of chilling stress in rice. Specifically, the application of nanoparticles (NPs) to the leaves of rice plants effectively decreased the harmful effects of chilling stress, demonstrating that NPs play a crucial role in controlling the plant’s response to cold temperatures by regulating the antioxidant enzymatic activity. The application of nanoparticles (ZnO, Fe_2_O_3_, TiO_2_, and CeO_2_) to the leaves of plants showed significant and diverse impacts on plant development and the production of chlorophyll. This ultimately resulted in increase of plant’s ability to scavenge reactive oxygen species (ROS) and enhance its antioxidant capability, particularly when exposed to cold stress. Hence, our research demonstrates the impact and mechanism of nanoparticles (ZnO, Fe_2_O_3_, TiO_2_, and CeO_2_) in mitigating the harmful effects of chilling stress on rice plants. This offers a viable approach for minimizing the adverse impacts of chilling stress on crops. The findings of this study demonstrate that the foliar application of ZnO, Fe_2_O_3_, TiO_2_, and CeO_2_ nanoparticles can effectively mitigate the adverse effects of chilling stress on rice plants. These nanoparticles enhance photosynthetic pigment retention, reduce oxidative stress damage, and increase antioxidant enzyme activities, thereby improving the plant’s overall stress resilience. However, a further genetic study is recommended to clearly understand the mechanism of these nanoparticles.

## Figures and Tables

**Table 1:** Plant height (cm), fresh weight (g/plant), dry weight (g/plant), root length (cm), Chlorophyll A, and Chlorophyll B of rice seedlings influenced by different nano-particle applications under chilling stress.

Nano-Particles (mg/L)	Plant Height (cm)	Fresh Weight (g/Plant)	Dry Weight (g/Plant)	Root Length (cm)	ChlA (µg/g FW)	Chl b (µg/g FW)
Zinc oxide(mg/L)						
CK	35.88 a	15.43 a	1.523 a	21.84 a	151.5 a	130.4 a
0	19.47 d	8.60 d	0.809 c	11.38 d	82.2 c	74.9 d
50	24.1 c	9.93 cd	0.922 c	14.09 c	94.4 c	87.1 cd
100	27.92 b	11.48 b	1.242 b	18.17 b	125.1 b	107.6 b
200	26.75 bc	11.13 bc	1.177 b	17.12 b	118.6 b	98.5 bc
LSD_(0.05)_	3.66	1.543	0.1135	2.628	12.83	14.86
Iron oxide(mg/L)						
CK	35.04 a	15.18 a	1.520 a	17.23 a	159.1 a	107.3 a
0	17.5 d	8.27 d	0.795 c	9.37 d	91.67 c	60.3 d
50	23.88 b	12.52 b	1.278 b	14.38 b	138.8 b	95.4 ab
75	23.34 b	11.05 bc	0.899 c	11.53 c	128.9 b	85.7 bc
100	20.0 c	10.71 c	0.871 c	10.18 cd	101.6 c	76.5 c
LSD_(0.05)_	1.92	1.72	0.1255	2.0393	12.28	14.83
Titanium oxide (mg/L)						
CK	35.88 a	16.68 a	1.508 a	22.7 a	144.8 a	128.2 a
0	16.98 d	9.46 c	0.795 c	12.508 b	75.7 d	69.7 c
50	27.74 b	11.32 b	1.278 b	12.825 b	128.4 b	93.1 b
75	25.95 bc	10.19 bc	0.899 c	12.64 b	118.6 b	82.9 bc
100	23.7 c	9.75 c	0.871 c	12.7 b	86.6 c	74.3 c
LSD_(0.05)_	2.92	1.22	0.1068	1.98	10.35	17.29
Cerium oxide (mg/L)						
CK	33.58 a	17.43 a	1.739 a	20.7 a	158.9 a	105.1 a
0	19.3 d	10.77 c	0.809 c	11.1 d	91.5 c	58.1 d
50	23.8 c	13.71 b	0.922 c	12.47 cd	101.5 c	74.4 c
75	27.6 b	15.1 b	1.308 b	15.67 b	138.7 b	93.2 ab
100	22.42 d	14.22 b	0.888 c	14.25 bc	128.9 b	83.6 c
LSD_(0.05)_	3.26	1.479	0.149	2.9	12.28	14.8
Cultivars						
LLY-7108	27.8 a	16.45 a	1.159 a	15.94 a	129.2 a	1 o3.1 a
LLY-32	24.8 b	13.68 bc	1.006 b	13.14 c	14.6 b	81.4 b
XZX-06	23.3 b	14.83 ab	1.048 b	14.3 b	123.9 a	96.01 a
Zhang-17	20.3 d	11.85 c	0.875 c	10.98 d	105.4 c	74.03 b
LSD	2.8	1.23	0.111	1.6	7.62	8.56

Means with the same lower-case letter are not significantly different among varieties; differences between treatment means were tested with Tukey’s test at 5% probability level.

## Data Availability

The datasets collected and/or analyzed in the present study are available from the corresponding author upon reasonable request.
